# The Outcome in Early Cases of Treatment of Subtrochanteric Fractures with Proximal Femur Locking Compression Plate

**DOI:** 10.5704/MOJ.1407.011

**Published:** 2014-07

**Authors:** U Gunadham, J Jampa, S Suntornsup, B Leewiriyaphun

**Affiliations:** Department of Orthopedics, Trang Regional Hospital, Trang, Thailand; Department of Orthopedics, Trang Regional Hospital, Trang, Thailand; Department of Orthopedics, Trang Regional Hospital, Trang, Thailand; Department of Orthopedics, Trang Regional Hospital, Trang, Thailand

## Abstract

The objective of this study was to evaluate the outcome in early treatment of subtrochanteric fractures with proximal femur locking compression plate (PF-LCP).The patients included in this study were those with subtrochanteric fractures (AO type 32A-C) treated with PF-LCP (Synthes) between Jan 2009 and Jun 2011. The patient characteristics and details of clinical conditions were obtained from records. Clinical and radiographic follow-ups were done at one, two, four and 6 months intervals, and at one year. The primary outcome studied included fracture union and functional ambulatory status. Twenty-six patients were included in the study, 19 of whom were male with a mean age of 42.4 years. Fourteen patients (53.9%) had sustained AO type 32B fractures, the majority in motor vehicle accidents. Twenty-two fractures (84.6%) achieved union, while sixpatients (23.1%) had complications such as broken plate, varus collapse, and broken screw. Four patients (15.4%) underwent a second operation. At the end of the follow-ups, 25 patients (96.2%) were community ambulators. We conclude that PF-LCP is an effective alternative treatment for subtrochanteric fractures when properly performed.

**Key Words:**

Subtrochateric fracture, Proximal Femur Locking Compression Plate (PF-LCP), Outcome, Complication, Cerclage wire

## Introduction

Subtrochanteric fractures account for 10-34% of all hip fractures ^1, 2^. They are due to high energy trauma in the young patients, while in elderly patients, they are often caused by low energy trauma in osteoporotic bone.^2^ Surgical treatment is the preferred method for subtrochanteric femoral fractures and a variety of implants are used. These implants fall into two main categories, intramedullary and extramedullary. Intramedullary fixation is associated with short operative time and minimal blood loss and has better biomechanical properties when compared with extramedullary fixation. However, they have their own technical difficulties and complications ^1, 3^.

Extramedullary devices such as dynamic condylar screws and 95° condylar blade-plates provide strong fixation in the cancellous bone of the neck and head with considerable rotational stability. Their disadvantages are longer operating time, technically demanding -, extensive devascularization, higher infection rate-, delayed weight bearing, medial instability, refracture after plate removal and surgical approach ^4^. The introduction of biologic, soft tissue sparing techniques has made plate fixation of femur a viable option. Indirect reduction and submuscular plating of subtrochanteric fractures produce good results ^5^.

The locking compression plate was introduced in the 21st century as a new implant that allowed angular-stable plate fixation for the treatment of complex comminuted and osteoporotic fractures in different anatomic regions ^6, 7^. Recently proximal femoral locking compression plate has been applied in the treatment of proximal femur fracture including subtrochanteric fracture. Locking plates have the advantage of allowing multiple angularstable fixation points into the proximal femur, while leaving a smaller ‘foot print’ by preserving more bone stock after implantation compared with the use of large proximal lag screws ^8^.

The Proximal Femur Locking Compression Plate (PFLCP) was introduced in Thailand around 2006 and had increased in popularity ever since. Initially it had been used mostly in complex and unstable intertrochanteric fractures and later in subtrochanteric fractures. Until recently, there have been few clinical studies of PF-LCP and the results are somewhat different ^9, 10^. From previous study, PF-LCP provides stable fixation with high union rate and few complications ^10, 11^, while some studies found high rate of failure even when performed by experienced surgeons ^9^. This study was conducted to review the early outcome of - cases of PF-LCP use in subtrochanteric fractures in a government hospital in Southern Thailand.

## Materials and Methods

This is a retrospective study of patients - aged 15 years or older, who had sustained subtrochanteric fractures (AO type 32A-C) and were treated with proximal femur locking compression plate (PF-LCP; Synthes) between Jan 2009 and Jun 2011. The patients with pathologic fractures (other than due to osteoporosis) were excluded. This study was approved by the institutional review board.

**Fig. 1The locking compression plate for the proximal femur is a precontoured, angular stable, with large fragment screw (7.3/5.0/4.5mm).**
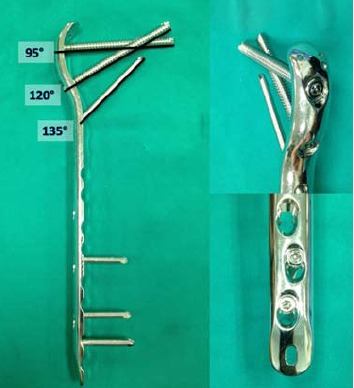


The PF-LCP is an angular-stable and limited contact plate specifically designed for treatment of complex, comminuted fractures of the per, inter and subtrochanteric femoral region. The plate is anatomically precontoured for the metaphysis of the proximal femur. The first two proximal threaded holes of the plate are designed for cannulated 7.3-mm locking head screws that are inserted at 95° and 120° in relation to the shaft of the femur. The third threaded round hole is for a cannulated 5.0-mm locking head screw that is inserted at the level of the calcar at 135° angle, and this screw intersects with the most proximal 7.3-mm screw, serving as a so-called “kickstand screw”. (Figure 1.)

The remaining screw holes, which range from 4 to 16 in the PF-LCP, are LCP-combi-holes that allow the placement of either a conventional (4.5 mm) or a locking head screw (5.0 mm) at the level of the shaft. The most distal hole allows the use of a Kirschner wire for temporary fixation to achieve correct positioning of the plate.

Surgery is performed with the patient supine under general anesthesia with traction on a fracture table. Through a standard lateral approach fluoroscopically guided reduction and distal submuscular plate advancement were feasible. Proximal fixation was obtained at least with the two most proximal screws (locking or conical/ nonlocking). A “kickstand screw” was used routinely. Cerclage wiring was used to aid fracture reduction in some cases. Distal fixation was obtained with a minimum of three locking screws.

Postoperatively, patients were allowed - toe-touch weight bearing for the first eight weeks, partial weight bearing after callus formation in radiographs, and weight as tolerated at 4 months. Clinical and radiographic followups were done at one, two, four and sixmonth intervals, and at one year.

Radiographs of hip and femur were reviewed to obtain the AO-OTA classification ^12^, fixation characteristics, medial contact stability, and the use of cerclage wire and kickstand screw. Patient charts were reviewed to collect baseline characteristics and clinical outcomes. Primary outcome included fracture union and functional ambulatory status. Pre- and post-fracture ambulations were classified based on standard definitions of community and household ambulators ^13^. All community ambulators were able to walk indoors and outdoors either independently or with assistive devices. Household ambulators were limited to indoor walking either independently or with assistive devices. Non-functional ambulators were either bedbound or limited to bed-to-chair transfers with assistance.

Statistical analyses were performed with Stata version 10.0 (StataCorp, College Station, TX). Continuous data were analyzed using mean ± standard deviation (SD) and unpaired t-test. Non-continuous data were analyzed using proportion, percentage, and Fisher’s exact test. The p-value < 0.05 was considered as a statistical significance.

## Results

Twenty-six consecutive patients were included in this study. Nineteen patients (73.1%) were male and 7 patients (26.9%) were female. Mean age was 42.4 ±23.2 years, mean weight was 64.7 ±12.4 kilograms, mean height was 165.1 ±8.6 centimeters, and mean BMI was 23.6 ±3.2 kg/m2. Ten patients (38.5%) were smokers. Fourteen patients (53.9%) had AO type 32B fracture, while eight patients (30.8%) and four patients (15.4%) were classified as AO type 32A and 32C respectively (Table I). Motor vehicle accidents - accounted for 19 patients (73.1%), low energy injuries - in four patients (15.4%), and falls from height - in three patients (11.5%). All patients were community ambulators prior to injury.

Average time to fixation was 7.7 ±3.9 days and average hospital stay was 14.5 ±5.6 days. Mean operative time was 109 ±22 minutes and mean blood loss was 619 ±276 ml. Cerclage wires were used in 12 patients (46.2%), while kickstand screws were used in 23 patients (88.5%). Nineteen patients (73.1%) achieved good medial buttress. Average of 2.7 proximal screws and 3.3 distal screws were applied (Table II).

Mean follow-ups was 11 ±6 months ( range 6 to 25 months). Twenty-two fractures (84.6%) achieved union, while six patients (23.1%) had complications. In two patients the plate had broken, two patients had varus collapse and one had broken screw and non-union of the fracture. Complications occurred in average of 4.3 ±2.1 months (range 2 to-7 months). Four patients (15.4%) underwent a second operation. Three patients had revision with PFLCP and one patient underwent revision with 135° angled blade plate. At the end of the follow-ups, 25 patients (96.2%) were community ambulators, while one patient was household ambulator (Table II).

Comparative analyses between group of patients with and without complication were performed. No statistical significance was found among gender, age, BMI, history of smoking, AO-OTA classification, the use of kickstand screw, or the presence of good medial buttress. However, it was discovered that cases with the use of cerclage wire had less complications than those without cerclage wire (0/12 in former group and 6/14 in latter group) with statistical significance (p-value = 0.01) (Table III).

## Discussion

Stable subtrochanteric fracture can be treated successfully with conventional implants, such as sliding hip screws, cephalomedullary nails, and angular blade plates. However, comminuted and unstable subtrochanteric fractures are challenging injuries that are prone to complications ^1,2^. Intramedullary device such as cephalomedullary nails showed increased fracture stability when compared to extramedullary devices. 14 In cadaveric study, cephalomedullary nail construct in the treatment of subtrochanteric fractures would be consistently superior biomechanically to either a PFLCP construct or a 95°angled blade plate construct when a considerable fracture gap persists. Furthermore, in the insertion of cephalomedullary nail a large portion of bone had to be removed from the proximal femur with unknown long-term effects ^15^.

The PF-LCP provided angular stability with greater degree of adjustment compared with angled blade plate, and offered the same variability while avoiding excessive bone removal compared with dynamic condylar screw ^16^. In order to prevent complication from subtrochanteric fracture, current trends are moving forward to biological fixation and subcutaneous insertion which PF-LCP ^5,11,17^. Until recently, there are few clinical results of PF-LCP from previous studies which would be expected -for a newly developed implant.^9, 10^ Our study attempted to collect data of early cases of PF-LCP in subtrochanteric fractures in a level-II trauma center in Southern Thailand.

From previous studies, the so-called kickstand screw played an important role in preventing varus collapse of the construct ^9, 18^. In most of our cases, the kickstand screw was utilized. In cases where the 95° screw was not in the most superior position, the kickstand screw could not be applied (Figure 2.) However, the absence of kickstand screw was not associated with complications in our study. Besides the use of fracture table, indirect reduction of the fracture can be achieved with the supplement of circumferential wire.^19, 20^ Unlike cephalomedullary nail, PF-LCP allows circumferential wire without additional incision. Cerclage wiring is an alternative technique to achieve reduction in difficult fractures ^21, 22^ From recent cadaveric study, cerclage wiring resulted in only minimal disruption of femoral blood supply ^23^. In this study, cerclage wiring was used to achieve near-anatomic reduction in some cases and had less complication statistically compared with the group without cerclage wire (Figure 3). This may be attributable to the near perfect anatomical reduction and good medial buttress of the fracture site as well.

The authors propose- that PF-LCP may be suitable implant in unstable and comminuted subtrochanteric fractures, fractures with extension to greater trochanter which non-feasible with cephalomedullary nail. In order to achieve promising result from PF-LCP, the surgeon should focus on adequate plate length, near-anatomical reduction with or without circumferential wire, good medial buttress of fracture site, use of kickstand screw, biologic friendly or subcutaneous insertion when possible. In developing countries such as Thailand, there are many considerations regarding implant choices. Although there is universal coverage through the National Health Care System, locking compression plates are among the high-cost implants which need cost-benefit considerations before application.

Limitation of this study are the small number of patients and less surgeon’s expertise with the implants which may affect the outcome. Further studies are needed to show the outcome and the effectiveness of this method of fixation in future cases after the have surgeons gained experiences with using the implants.

**Table I : Patient Characteristics:**
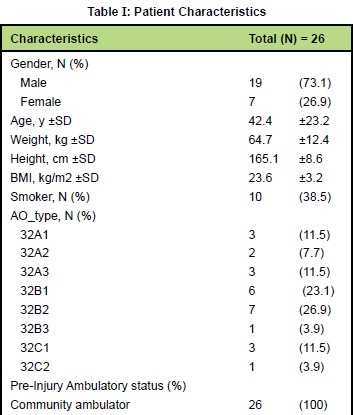


**Table 2 : Outcomes:**
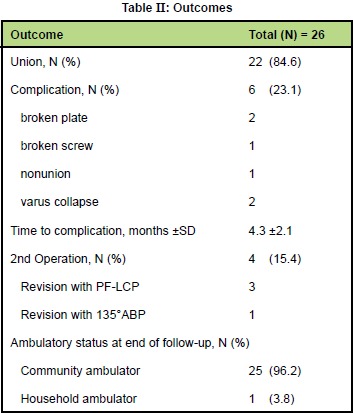


**Table 3 : Comparative Analyses:**
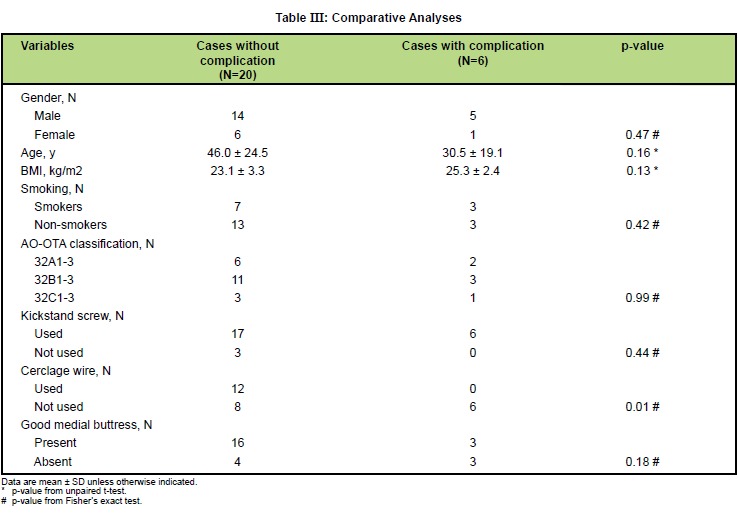


**Fig. 2: Example of case which the “kickstand” screw was not applied. Pre-operative (A), Immediate postoperative (B), 2-month post-operative (C), 4-month post-operative (D).**
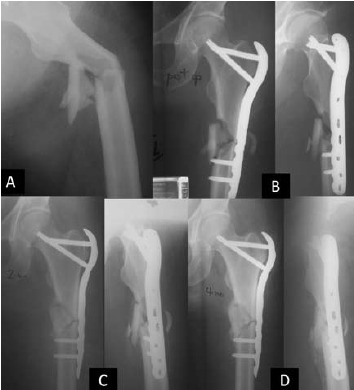


**Fig. 3:Near-anatomic reduction of unstable subtrochanteric fracture by circumferential wire application. Pre-operative (A,B), Immediate post-operative (C), 6-month post-operative (D).**
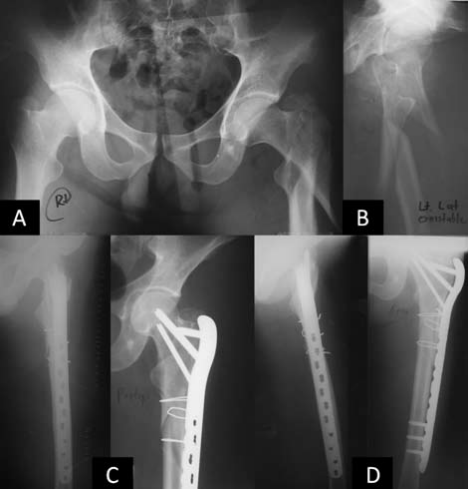


## Conclusion

The PF-LCP represents a feasible alternative for the treatment of subtrochanteric fractures when properly performed. Further clinical studies are necessary to show its role in the treatment of these fractures.

## Conflict of interest statement declarations

The authors did not receive payments, other benefits, or a commitment or agreement to provide such benefits from a commercial entity.
